# The Centenary of the Leprosy Relief Association (Lepra)—a moment for celebration and reflection^†^

**DOI:** 10.1093/trstmh/trad096

**Published:** 2024-01-25

**Authors:** Irene Allen, Diana N Lockwood, Roderick J Hay

**Affiliations:** Lepra, Colchester, CO1 1TG, UK; Department of Infectious Diseases, London School of Hygiene and Tropical Medicine, London WC1E 7HT, UK; King's College London, St John's Institute of Dermatology, London SE1 7EP, UK

**Keywords:** centenary, history, Lepra, leprosy

## Abstract

The year 2024 is the Centenary of the foundation of the Leprosy Relief Association (Lepra), formerly the British Empire Leprosy Relief Association (BELRA). The name of the organization changed to the LEProsy Relief Association (LEPRA) in 1976 but has been known as Lepra since 2008. Over the years it has worked closely with members and office holders of the Royal Society of Tropical Medicine and Hygiene. Its work has encompassed activities from the earliest initiatives to ensure appropriate living conditions for those with the disease to the development of leprosy chemotherapy. However, this has now evolved into a strong partnership between the UK- and India-based Lepra hubs, which are carrying out research and public health initiatives ranging from elimination of prejudice against those with leprosy to adopting the recently launched WHO programme for skin NTDs to facilitate integrated control and management regimens. The fight against leprosy has always been a partnership between a wide variety of disease-specific NGOs, health-care workers and international health agencies. The story of Lepra illustrates the central role of these partnerships and national as well as international collaboration.

## Introduction

Victorian Britain saw the renaissance of a widespread interest in the ‘heroic’ ideal. Admiration for truly unselfish acts was celebrated and publicized. So, the life and fate of a Belgian Roman Catholic priest, Father Damien, sanctified in 2007, whose devoted care and responsibility for the enforced inmates of the Hawaiian leprosy settlement on the island of Molokai, struck a chord with public opinion. This sentiment was magnified greatly as, in setting about his mission, Damien himself developed leprosy.^1^ A British movement to support Damien's work, the Father Damien Foundation, was formed to raise funds under the royal patronage of Edward, Prince of Wales, but its work was temporarily halted by the news of the priest's death from advanced lepromatous leprosy in 1889. Donations to the fund continued and the money raised was used to improve knowledge of the disease and training in Britain and India and to provide a memorial to Father Damien.^2^ Other charitable organizations focusing on this disease were formed in the late nineteenth century. These included the St Francis Leprosy Guild, a Roman Catholic charity established by Kate Marsden, an energetic British nurse whose mission to the people with leprosy in Siberia was much admired,^3^ and the Mission to Lepers, a broad church-based organization with a strong missionary-led programme of care.^4^ The other British organization dedicated to charitable work and leprosy is the English and Scottish Grand Priory of the Order of Saint Lazarus, whose foundation dates back to the eleventh century.

## The creation of the British Empire Leprosy Relief Association (BELRA)

The death of Queen Victoria in 1901 and the ascent of the Prince to the throne as Edward VII, followed by wars in South Africa and then in Europe meant that little further public action was taken to improve the lot of those suffering from a disease that had largely been forgotten, but whose effects were all too apparent to doctors, nurses and missionaries working in the resource-poor areas of the British Empire. In the early 1920s a new charitable undertaking to improve the lives of those with leprosy and to carry out research into the disease was initiated with the creation of the British Empire Leprosy Relief Association (BELRA). This was the logical consequence of a growing medical and public debate about the need to improve understanding of this disease and to garner medical support and promote compassion for those with the condition. The association was launched at a meeting in Mansion House, London presided over by the Lord Major on 31 January 1924. Opening donations had been made by King George V, and by his eldest son, the Prince of Wales, who became Patron. At this time the published estimate of case numbers of those with leprosy in the British Empire was about 300 000, of whom 102 000 lived in India^5^; other areas included in this total were the West Indies, West Africa (Ghana, Nigeria) and East Africa (Zambia, Kenya, Malawi, Uganda and Zimbabwe). These figures grossly underestimated the extent of the disease. The aims of the new association were set out in the opening speeches. ‘The British Empire Leprosy Relief Association will work largely through existing agencies in distributing drugs, information regarding their use and the latest medical advances, by helping sound schemes of segregation combined with the latest treatment, training the staffs of leper institutions, and supporting research for still further improving the treatment, which fails to completely clear up many advanced cases; in short, to coordinate treatment and research.’^6^

The three individuals involved in the establishment of BELRA were Sir Leonard Rogers, the Reverend Frank Oldrieve, secretary of the Mission to Lepers, and Sir Frank Carter, a businessman with philanthropic interests, based in Calcutta. Leonard Rogers was Professor of Pathology in Calcutta. His previous best-known work had been on amoebic dysentery, but he became aware of the challenges of leprosy, and was interested in the use of chaulmoogra oil from the fruit of the tree *Gynocardia odorata*, to treat the disease. This had been used for many years as a natural medicine for treating leprosy in India and other countries in the East, but in 1854 a report published in the medical press, and largely ignored at the time, discussed and demonstrated its use in leprosy patients in India.^7^ The chief problem with giving chaulmoogra oil as a medication was that it was highly irritant if taken by mouth, leading to nausea and vomiting and, if injected, caused both pain and local inflammation. Despite this it was given to patients. But with the development of medicinal chemistry in the late nineteenth century scientists sought to isolate the active ingredients, hoping that they would prove less toxic but more effective. Some of these chemicals, chaulmoogric, hypnocarpic and gynocardic acids, were some of the more tolerable derivatives under investigation. Rogers had seen the dire state of patients with leprosy in India and his interest was aroused by this challenge. He experimented with using sodium salts of the essential acids and found that they were tolerated better and did not have the irritant side effects of unpurified chaulmoogra oil extract.^8^ Aware of his interest in the disease Frank Oldrieve approached Rogers with a proposal to create a new charitable organization for the relief of leprosy, which then led to the recruitment of the third member of the founding team, Frank Carter, who brought financial expertise to the endeavour. They finalized the details of the new charity in July 1923 at India House and launched BELRA in January 1924 at Mansion House.

## The promotion of care for leprosy patients

Sir Leonard Rogers became an outstanding advocate of the cause of leprosy, not just through his work on chaulmoogra, but through his advocacy for the proper care of those with the disease. He had deep reservations about the strategy of enforced isolation of leprosy patients, believing, pragmatically, that this was a serious obstacle to obtaining the confidence of those with the disease. He discussed this in 1923 advocating the use of open ‘colonies’ with plenty of space and land for families as opposed to what were known as asyla, which he regarded as restrictive and prison-like.^9^ While recognizing the contagious nature of leprosy he also knew that simple exposure to the organism alone would not necessarily lead to established infection. His views crystalized further as better treatments became available. As he stated in 1928 in presenting his annual report on the work of BELRA^10^: ‘A decade ago only segregation was available, but, in addition to being infinitely more expensive than our present dispensary system, it inevitably resulted in the poor lepers hiding themselves until too advanced to profit much by treatment, and in their infecting others before they were discovered and isolated, so it may possibly do more harm than good now an effective treatment for early cases is available. Where compulsory segregation is already in force, we do not advise its immediate abolition, but that all newly discovered lepers should be examined by a small board of expert medical men, with power to permit early uninfected cases to be treated as outpatients at dispensaries instead of being segregated, so as to attract for treatment the early amenable cases .’ His views contrasted with prevailing medical thinking and nervous and unsympathetic colonial administrations that were concerned about containing the spread of the disease. Despite this, subsequent work on improving the care of those with leprosy included the construction of leprosaria centred on communal care and the provision, where possible, of occupational activities to provide a measure of independence, as well as financial support. An example was the Sungai Buloh leprosarium in Malaysia built in 1930 covering 562 acres and this locality reflects, to this day, the wider ambition as part of the original site is now a nursery garden complex.^11^ Sir Leonard Rogers was subsequently elected President of the Royal Society of Tropical Medicine and Hygiene (RSTMH) in 1933. He continued his interest in leprosy until his death in 1962; he retired to his birthplace, Devon, but continued to correspond and address areas of interest.^12^ The links between the RSTMH and BELRA were deep but were not restricted to Rogers.

The early years of BELRA in the post First World War era and the subsequent financial depression were not easy and were marked by financial stringency. The organization, however, also started to open overseas branches, the first of which was in India in 1925, but subsequently extended the work to sub-Saharan Africa and the Caribbean. The generous financial intervention by a group of Indian rulers, a large donation known as ‘the Maharajah's gift’, provided much needed support. BELRA also attracted the interest of the Reverend Phillip Thomas Byard Clayton (‘Tubby’ Clayton), a far-sighted man who founded ToC H during the First World War for the support of servicemen, which evolved into a Christian charity for the demobbed and others suffering the consequences of post-war austerity; Clayton recruited volunteers to help with work for BELRA in different countries and to raise finance for the organization.^13^ As it developed, the work of the British Empire Leprosy Relief Association was divided into four sections: (1) the direct grants to institutions in the field where definite preventive and curative work was being done; (2) the supply of drugs and instruments for treatment; (3) the distribution of literature to keep workers up to date in all aspects of the subject; and (4) research work. The Association became a clearing house for receiving and disseminating information, so that successful methods adopted in one place might be quickly used in other places.^14^ The journal *Leprosy Review* came into being in order to supply the need for information and to publicize research. BELRA also worked closely with other leprosy charities both in service delivery as well as research.

## The treatment of leprosy—the new vision

The work of BELRA was also marked by its promotion of new therapies. Purified derivatives of chaulmoogra oil were used as treatments throughout the 1920s and 1930s but they were still associated with side effects and although remissions occurred there were both treatment failures and relapses after apparent recovery. However, in the early 1940s experiments with a sulfonamide derivative, promin, were undertaken by the US Public Health Service at the Federal Leprosy Hospital, Carville, USA.^15^ This was best given intravenously and led to clear improvement in the clinical state of leprosy, although side effects were both common and severe. In 1944 Dr Ernest Muir carried out studies in Chacachacara Leprosarium, Trinidad with a different derivative, diasone, which produced clear improvements in the clinical state of lepromatous leprosy patients.^16^ Muir was the Medical Secretary of BELRA. The first clinical trials of a further sulfonamide derivative, dapsone, were carried out by an RSTMH member and, also, medical secretary of BELRA, Robert Greenhill Cochrane, in Madras.^17^ He compared different sulfone derivates and in 11 patients he used the new compound diaminodiphenyl-sulfone (dapsone). When dapsone was given by injection anaemia and other side effects developed in patients. The work was taken forward under the auspices of BELRA in Nigeria where Dr John Lowe treated patients with oral dapsone since this was the active compound of many of the sulfones.^18^ The results were impressive. Of 15 patients with tuberculoid leprosy all showed subsidence of activity within six months and 62% of the lepromatous cases showed significant clinical improvement. The trial showed that leprosy patients could be cured and return to their former lives, free of the infection although often still having residual signs of leprosy with trophic skin changes, ulcers, and neurological damage. Some, after years of isolation, remained in the old leprosy hospitals that had been their homes for much of their lives. Cochrane was President of the International Leprosy Association from 1965 to 1968. The Royal Society of Tropical Medicine and Hygiene also created and administered the Robert Cochrane Fund for Leprosy to provide bursaries to young leprosy researchers in his honour.

The rise of dapsone resistance, an inevitable consequence of treating a slowly growing bacterial infection over long periods of time, was also the focus of concern of RSTMH members such as Stanley Browne. Browne, later a Trustee of Lepra, started his career in tropical medicine working as a medical missionary in Yakusu in the Congo in 1939 and, struck by the high prevalence of the disease, helped to create a leprosarium at the site. He promoted using the anti-bacterial clofazimine in the treatment of leprosy to slow the development of drug resistance. His skills extended from treatment to history. He became President of the Royal Society of Tropical Medicine and Hygiene in 1977.^19^ Browne and others were concerned by the spread of dapsone resistance and they promoted the development of the multidrug therapy regimen, which was formally recommended by WHO in 1981. Although there have been changes in composition and duration of multidrug treatment regimens since then dapsone, rifampicin and clofazimine are still used today and form the core of leprosy treatment packs.^20^

## BELRA to LEPRA and Leprosy Society India

After the Second World War the creation of the new states of India and Pakistan as well as other postcolonial independence movements changed the status of BELRA and its work in India ceased. It was renamed LEPRA, the LEProsy Relief Association. It became a member of the International LEProsy Federation (ILEP) in 1976. For 40 years the association worked independently of Indian organizations. But in 1989 LEPRA's Indian colleagues set up a treatment project using multidrug therapy in Karnataka State. It became clear that in the interests of providing smooth collaborative work, an independent Indian organization with devolved responsibility should be formed and on 3 August 1989, LEPRA Society India was established as a separate but linked body.^20^ So, the two, LEPRA and Leprosy (LEPRA) Society India, came together again in a closely allied partnership working on common tasks, since 2008 known as Lepra. The LEPRA Society India was led initially by Dr K Desikan who had a clinical medicine and pathology background and brought a holistic approach to patient care. This alliance continues in close collaboration to this day. Lepra India has also carried out important work in leprosy control and surveillance. Centring its activities in different states and districts including Andhra Pradesh, Bihar, Chhattisgarh, Delhi, Jharkhand, Madhya Pradesh, Odisha, Telangana, Uttar Pradesh, it has also led in developing integrated pathways for NTD management, e.g. with lymphatic filariasis.^21^ The television programme, Blue Peter, generously contributed to this effort by launching the Christmas appeal in 1996 to provide funds to create a research centre for leprosy at the Indian headquarters in Hyderabad—the Blue Peter Public Health and Research Centre or BPHRC, with Dr Sujai Suneetha as director. The centre took part in a number of projects including a large prospective study to identify risk factors for developing nerve damage^22^ and leprosy reactions.^23^ The BPHRC was led by the immunologist Professor Indira Nath in the mid 2000s. She did important work on identifying immunological processes in leprosy. The centre is also currently studying the development of drug resistance^24^ and the management of leprosy ulcers^25^ under the guidance of Dr Aparna Srikantam.

## Other projects

One of LEPRA's other interests was the development of large public health programmes for control of and research into leprosy. The first intervention of this type was the creation in 1953 of a leprosy research centre in Eastern Africa at the Itesio Leprosarium in Kenya.^26^ It now forms part of the Kenya Medical Research Institute. A further centre in sub-Saharan Africa was also planned and, after considerable discussion, a suitable location was found where a project to study the control of leprosy could be established. The site selected was an area covering 2000 square miles near Blantyre in Malawi, with a population of 1.3 million recorded in the 1966 census, and an estimated 10 000 leprosy sufferers with a leprosy prevalence of at least 10 per 1000.^27^ This was an area of moderate endemicity, but as well as new cases there was backlog of old cases that had to be treated. The objective was to ensure that all leprosy cases, registered as well as those yet to be identified, would be treated within a 10-year span. The project was based on a centre that was to be built in the grounds of the Queen Elizabeth Hospital in Blantyre, but in addition there would be enough vehicles and staff to make treatment available at points within a range of achievable access for all patients. Funds were available from the Brown Memorial Trust in Malawi to augment the £500 000 that LEPRA would have to raise for the project. Within four years almost 10 000 patients had been registered and treated. The annual cost of treating each patient was estimated to be just over £3.^27^ Work in Malawi led to many publications on the diagnosis, treatment and outcomes of leprosy patients. However, a later and key development was the Karonga project led by Paul Fine. Amongst other important contributions it studied the effect of the BCG vaccine on the development of leprosy showing a post-immunisation protective effect of at least 50% in this environment, and provided new insights into the spread of leprosy.^28^ The study also showed that adding *M. leprae* to BCG did not enhance protection against disease, an important negative finding. The centre carried out important work on other aspects of leprosy including the risk of recurrence, genetic susceptibility and the impact of disability.^29^ It has now become the Malawi Epidemiological and Intervention Research Unit, which has a large staff and carries out longitudinal studies on long-term health conditions in low-income Africans.

LEPRA also engaged in public advocacy. In 1965, the film ‘The Name of the Cloud is Ignorance’, made free of charge for LEPRA by Richard Bigham, about its work in India, was presented at the Venice Film Festival and won two awards; the next year it was entered for the XIX International Film Festival at Salerno where it won an award for the best documentary film. in 1975 Richard Bigham made a second film for LEPRA, about the work in Malawi this time, entitled ‘Outpatients not Outcasts’, which was in colour and ran for 30 minutes.^30^

## Leprosy Review

BELRA also expanded the focus on leprosy through publications. Frank Oldrieve had started this work through articles in an embryonic publication, Leprosy Notes, but Dr Cochrane, who became involved with LEPRA because of his medical expertise, initiated the change that transformed the Notes into an authoritative medical journal. The quality of the contributions being received was sufficient to warrant this, and he felt that BELRA's status would be enhanced. Although Sir Leonard was initially against this idea (‘We are not in a position to start a scientific journal’), it was decided that Leprosy Notes would be published as *Leprosy Review* and subtitled ‘The Journal of the British Empire Leprosy Relief Association’. The journal is now the major peer-reviewed journal for leprosy work, since the *International Journal of Leprosy* stopped publication in 2005, because of the decline in research on leprosy with the perception that leprosy was eliminated as a public health problem.

## The future

As it celebrates its centenary, Lepra remains a highly active organization. It works with other partner organizations from ILEP as well as with individual clinicians and other health-care sector workers. Leprosy has not been eliminated and indeed there are over 200 000 new cases recorded by WHO annually, with India, Brazil and Indonesia reporting the largest numbers of cases. New initiatives such as the millennium target of elimination of leprosy, defined as a reduction of the case numbers to below 1:10 000, have come and gone and, while not rising, case numbers are still substantial; the major challenge—to find and treat the many cases that have not been identified by existing clinical services—remains. Active case finding is a key strategy for leprosy control. But there are other new initiatives. For instance schemes to reduce the prevalence of infection in contacts of infected cases with single dose rifampicin therapy have been introduced in some countries.^31^ Equally, targets such as an accurate point of care diagnostic laboratory test are still a distant dream. There is, however, exciting new work on the development of novel anti-leprosy and anti-leprosy reaction drug regimens and work on combatting the stigma and disability caused by the disease. This work addresses the impact of the disease on the most vulnerable, namely women and children. The initiation of the skin NTD programme by WHO, which aims to provide a framework for integrating a group of tropical diseases that present on the body surface and are subject to common patterns of disability and to discrimination, has provided a boost to this work.^32^ Lepra has refocussed its mission, in collaboration with Indian colleagues, on work in India and Bangladesh and has broadened its approach along current lines of thinking about NTDs in leprosy and lymphatic filariasis. It keeps a watching brief on work in sub-Saharan Africa and remains a global advocate for patients, research and improved care. Lepra has also promoted the New Face for Leprosy project, which provides evidence through photographs and personal statements of the courage and optimism of those with the disease.^33^

This centenary year is an important occasion for celebration. Yet it is profoundly thought-provoking that, one hundred years after its inauguration, there is still a need for Lepra and other NGOs and charities focusing on this old, treatable—yet still unconquered—infection.

## Data Availability

None.
